# Transgenic tomatoes expressing the 6F peptide and ezetimibe prevent diet-induced increases of IFN-β and cholesterol 25-hydroxylase in jejunum

**DOI:** 10.1194/jlr.M076554

**Published:** 2017-06-07

**Authors:** Pallavi Mukherjee, Greg Hough, Arnab Chattopadhyay, Mohamad Navab, Hannah R. Fogelman, David Meriwether, Kevin Williams, Steven Bensinger, Travis Moller, Kym F. Faull, Aldons J. Lusis, M. Luisa Iruela-Arispe, Kristina I. Bostrom, Peter Tontonoz, Srinivasa T. Reddy, Alan M. Fogelman

**Affiliations:** Departments of Medicine,* David Geffen School of Medicine at UCLA, Los Angeles, CA; Molecular and Medical Pharmacology,† David Geffen School of Medicine at UCLA, Los Angeles, CA; Human Genetics,** David Geffen School of Medicine at UCLA, Los Angeles, CA; Microbiology, Immunology, and Molecular Genetics,†† David Geffen School of Medicine at UCLA, Los Angeles, CA; Pathology and Laboratory Medicine,††† David Geffen School of Medicine at UCLA, Los Angeles, CA; Obstetrics and Gynecology,§§§ David Geffen School of Medicine at UCLA, Los Angeles, CA; Semel Institute for Neuroscience and Human Behavior,§ David Geffen School of Medicine at UCLA, Los Angeles, CA; Department of Molecular, Cell, and Developmental Biology,§§ College of Letters and Sciences, University of California, Los Angeles, CA; Howard Hughes Medical Institute,*** Los Angeles, CA

**Keywords:** apolipoprotein A-I mimetic peptides, ather­osclerosis, hyperlipidemia, oxysterols

## Abstract

Feeding LDL receptor (LDLR)-null mice a Western diet (WD) increased the expression of IFN-β in jejunum as determined by quantitative RT-PCR (RT-qPCR), immunohistochemistry (IHC), and ELISA (all *P* < 0.0001). WD also increased the expression of cholesterol 25-hydroxylase (CH25H) as measured by RT-qPCR (*P* < 0.0001), IHC (*P* = 0.0019), and ELISA (*P* < 0.0001), resulting in increased levels of 25-hydroxycholesterol (25-OHC) in jejunum as determined by LC-MS/MS (*P* < 0.0001). Adding ezetimibe at 10 mg/kg/day or adding a concentrate of transgenic tomatoes expressing the 6F peptide (Tg6F) at 0.06% by weight of diet substantially ameliorated these changes. Adding either ezetimibe or Tg6F to WD also ameliorated WD-induced changes in plasma lipids, serum amyloid A, and HDL cholesterol. Adding the same doses of ezetimibe and Tg6F together to WD (combined formulation) was generally more efficacious compared with adding either agent alone. Surprisingly, adding ezetimibe during the preparation of Tg6F, but before addition to WD, was more effective than the combined formulation for all parameters measured in jejunum (*P* = 0.0329 to *P* < 0.0001). We conclude the following: *i*) WD induces IFN-β, CH25H, and 25-OHC in jejunum; and *ii*) Tg6F and ezetimibe partially ameliorate WD-induced inflammation by preventing WD-induced increases in IFN-β, CH25H, and 25-OHC.

Studies in animals (1–4) of an apoA-I mimetic peptide synthesized from all D-amino acids (D-4F) that was developed in our laboratory led to studies in humans (5). At high doses (but not at low doses), and despite achieving very low plasma levels of peptide at the high doses, oral D-4F improved HDL antiinflammatory properties without altering plasma cholesterol levels (5). Administration by injection into humans of low doses of 4F peptide (peptide Ac-D-W-F-K-A-F-Y-D-K-V-A-E-K-F-K-E-A-F-NH_2_) synthesized from all L-amino acids yielded high plasma levels of peptide, but did not improve HDL antiinflammatory properties (6). We performed additional studies in mice to resolve the discrepancy between these two studies in humans (5, 6). These mouse studies demonstrated that the dose of peptide administered, and not the peptide plasma level, determined the efficacy, regardless of the route of administration (7, 8). These studies (7, 8) also demonstrated that the dose required for efficacy was far above the highest dose tested in the human clinical trials that did not demonstrate efficacy (6). Subsequently, we found that intravenous administration of either D-4F or L-4F in mice resulted in a remarkable targeting of the peptide to the proximal small intestine (the duodenum and jejunum), followed by transport of the peptide together with cholesterol into the intestinal lumen by the process of transintestinal cholesterol efflux (TICE) (9). Based on these data, we concluded that the 4F peptide functions as a modulator of the TICE pathway and suggested that the antiinflammatory functions of 4F may be a partial consequence of the codependent intestinal transport of both 4F and cholesterol (9).

In contrast to the 4F peptide, a related peptide with two additional phenylalanine residues on the hydrophobic face of the class A amphipathic helix [6F (peptide D-W-L-K-A-F-Y-D-K-F-F-E-K-F-K-E-F-F without end blocking groups)] was expressed as a transgene in tomatoes and fed as freeze-dried tomato powder (Tg6F). Tg6F (unlike 4F) reduced plasma cholesterol, triglycerides, and serum amyloid A (SAA) levels; increased plasma HDL cholesterol levels and paraoxonase activity; and decreased aortic atherosclerosis in mice (10).

Nakano et al. (11) reported that ezetimibe promotes TICE by targeting the Niemann-Pick C1-like 1 (NPC1L1) protein, which blocks internalization of cholesterol from the brush border membrane. As a result, cholesterol in the brush border exits by diffusion into the lumen of the small intestine (11), or ABCG5/G8 pumps it into the lumen of the small intestine (12). Jakulj et al. (13) reported that TICE is active in both mice and humans and controls ezetimibe-induced fecal neutral sterol excretion.

Both 4F and ezetimibe have been reported to work in the small intestine (9–11, 13), and both have been reported to promote TICE (9, 11, 13). Both ezetimibe and Tg6F lower plasma cholesterol levels, and Tg6F acts in the small intestine (10, 14). Therefore, it seemed reasonable to determine whether ezetimibe would enhance the amelioration of dyslipidemia and systemic inflammation by Tg6F. We report here that that is indeed the case. We also report a novel method for preparing Tg6F and ezetimibe for oral administration that is substantially better than adding them separately to a Western diet (WD) and feeding them as a combined formulation to LDL receptor (LDLR)-null mice. Finally, we show that Tg6F and ezetimibe prevent WD-induced increases in *i*) IFN-β, *ii*) cholesterol 25-hydroxylase (CH25H), and *iii*) 25-hydroxycholesterol (25-OHC) in jejunum.

## MATERIALS AND METHODS

### Materials

Transgenic tomatoes expressing the 6F peptide (Tg6F) were grown and processed as described (10). Ezetimibe was from Cayman Chemical Co. (Ann Arbor, MI, catalog no. 16331). Recombinant mouse IFN-β was from R&D Systems (Minneapolis, MN, catalog no. 8234-MB). Antibody to IFN-β was from Thermo Scientific (Waltham, MA, catalog no. PA5-20390). Antibody to CH25H was from Bioss Antibodies (Woburn, MA, catalog no. bs-6480R). Unless otherwise stated, all other materials were from sources previously described (14).

### Mice and diets

LDLR-null mice originally purchased from Jackson Laboratories on a C57BL/6J background were from the breeding colony of the Department of Laboratory and Animal Medicine at the David Geffen School of Medicine at UCLA. The gender and age of the mice are in each figure legend. The mice were maintained on standard mouse chow (Ralston Purina) before being switched to WD (Teklad, Harlan, catalog no. TD88137).

#### Single agents and the combined formulation.

In experiments in which Tg6F or ezetimibe was administered as a single agent, the Tg6F concentrate was added to WD at 0.06% by weight of diet (15) or ezetimibe was added to WD to give a final dose of 10 mg per kg of body weight per day. Tg6F concentrate or ezetimibe was mixed into the diets as described (15). In experiments in which the Tg6F concentrate was added to WD together with ezetimibe, each was added separately to WD at the same dose as used for the single agents, and each was mixed into the diet as described (15). Hereafter, we refer to the addition to WD of the Tg6F concentrate together with ezetimibe as the “combined formulation” to distinguish it from the “novel method” described below.

#### A novel method.

In some experiments in which Tg6F and ezetimibe were both administered, instead of adding Tg6F and ezetimibe separately to WD as described above for the combined formulation, ezetimibe was added during the preparation of the Tg6F concentrate. As previously described (14, 15), the concentrate was prepared by incubating the freeze-dried transgenic 6F tomato powder overnight in ethyl acetate with 5% acetic acid. During the incubation, tomato polyphenols and the 6F peptide partitioned into the ethyl acetate phase. The remaining solids were removed by centrifugation, leaving the tomato polyphenols and the 6F peptide in the supernatant. In the novel method, an amount of ezetimibe was added to this supernatant sufficient to provide 10 mg per kg of body weight per day of ezetimibe in the final preparation. After addition of ezetimibe to the supernatant, the mixture was incubated at room temperature (RT) with gentle mixing for periods ranging from 2 h to overnight. It was found that adding ezetimibe to the ethyl acetate-acetic acid supernatant and incubating for 2 h was as effective as allowing the incubation to continue overnight (data not shown). Therefore, a 2 h incubation was used for all experiments reported here. After the 2 h incubation, the ethyl acetate was removed as described (14, 15), and the resulting powder was resuspended in water and freeze-dried (14). The final freeze-dried tomato powder containing the 6F peptide together with ezetimibe is hereafter referred to as the novel method to distinguish it from the combined formulation. The final novel method freeze-dried tomato powder was mixed into the diet as described (14, 15) and was fed to the mice to give them the same dose each day of Tg6F (0.06% by weight of diet) and ezetimibe (10 mg per kg of body weight), as was the case when the agents were administered singly or in the combined formulation. Each day, the mice ate all of the food administered for each condition; there was no difference in food consumption between groups.

At the end of the treatment periods, the mice were fasted overnight in clean cages with new bedding, and, after blood collection for plasma determinations, after an overdose of isoflurane anesthesia, the mice were perfused extensively with cold saline to remove blood prior to harvesting the duodenum or jejunum (10, 16). The Animal Research Committee at UCLA approved all mouse studies.

### Assays

#### Detection of IFN-β or CH25H protein by immunohistochemistry.

After an overdose of isoflurane anesthesia, the mice were perfused extensively with cold saline to remove blood before harvesting the jejunum. Five mice (chosen at random) from each treatment group were used, and five segments of jejunum from each mouse were analyzed. Immunohistochemistry (IHC) was performed by the Immunohistochemistry Core in the Department of Pathology at David Geffen School of Medicine at UCLA. The jejunum segments were fixed in 10% neutral buffered formalin (pH 7.4) at physiological pressure at RT overnight. The fixed segments were thoroughly washed with distilled water and transferred to 70% ethanol, followed by embedding in paraffin and sectioning. For staining, the slides were placed in xylene to remove paraffin and then placed in a series of ethanol solutions. After a wash in tap water, the slides were incubated in 3% hydrogen peroxide/methanol solution for 10 min. After a wash in distilled water, the slides were incubated for 25 min in citrate buffer (pH 6.0) at 95°C by using a Decloaking Chamber NxGen (Biocare Medical, catalog no. DC2012). The slides were then brought to RT, rinsed in PBS containing 0.05% Tween-20 (PBST), and then incubated with anti-IFN-β antibody (Thermo Scientific, catalog no. PA5-20390) at a dilution of 1:300 overnight at 4°C or incubated with anti-CH25H antibody (Bioss Antibodies, catalog no. bs-6480R) at a dilution of 1:100 overnight at 4°C. In the next step, the slides were rinsed with PBST and incubated with Dako EnVision plus System-HRP Labeled Polymer Anti-Rabbit (Dako, catalog no. K4003) at RT for 30 min. After a rinse with PBST, the slides were incubated with 3,3′-diaminobenzidine for visualization. Subsequently, the slides were washed in tap water, counterstained with Harris’ hematoxylin, dehydrated in ethanol, and mounted with medium. Photomicrographs of the sections were captured by using an Olympus BX51 microscope and the application Q Capture 7.0 (Q Imaging, Inc.). Randomly selected fields were quantified for each sample, and the ratio of the stain signal to the villus area of the jejunum was determined by using ImagePro Plus 7 (Media Cybernetics). Three or four random fields on each of the five sections taken from each of the five jejunums were analyzed at 20× magnification as previously described (14). The data are expressed as the percent of villus area stained for IFN-β or CH25H for each condition. Negative controls were sections that were incubated with the secondary antibody alone (i.e., these sections did not receive the primary antibody).

#### Preparation of enterocytes.

After 2 weeks of treatment, the mice were fasted overnight, blood was removed under mild anesthesia, and the mice were euthanized. The abdomen was opened to completely expose its contents. The upper portion of the stomach was cut open, and a mouse feeding needle was inserted into the duodenum and fixed in place by ligation. A small opening was made at the end of the ileum to provide an outlet for the wash solution. Cold PBS was infused to wash the intestine in situ, while leaving the villi and the enterocytes intact and avoiding perforation of the intestine. Additionally, this resulted in an effective separation of the outer fat layers from the intestine. The jejunum was isolated and carefully inverted. Fifty milligram sections of jejunum tissue were removed, both ends were ligated, and the jejunum was frozen at −80°C. At the time of isolation of enterocytes, the frozen jejunum was incubated with buffer A (1.5 mM KCl, 96 mM NaCl, 8 mM KH_2_PO_4_, 27 mM sodium citrate, 5.6 mM Na_2_HPO, pH 7.3, 0.1 mM PMSF, and 1 mM benzamidine) for 15 min at 37°C in a shaking water bath. Buffer A was then replaced with buffer B (2.7 mM KCl, 137 mM NaCl, 1.5 mM EDTA, 1.5 mM KH_2_PO_4_, 8.1 mM Na_2_HPO_4_, pH 7.4, 0.5 mM DTT, 0.1 mM PMSF, and 1 mM benzamidine) and incubated for 30 min at 37°C with shaking. Enterocytes were released by gently vortexing the tube. The remaining tissue was removed and discarded, and the tube was centrifuged at 1,500 *g* for 10 min at 4°C to pellet the enterocytes. The supernatant was removed and discarded.

To determine the purity of the isolated enterocytes, the cells were incubated with Zombie Aqua (Biolegend, catalog no. 423101) together with anti-mouse CD326 (Ep-CAM) antibody (eBiosciences, catalog no. 118213) at 1:100 dilution. After 45 min, the cells were washed twice with fluorescence-activated cell sorting (FACS) buffer (PBS + 5% FBS). After a short spin, the cells were suspended in 300 μl of ice-cold PBS buffer and transferred to tubes for FACS analysis. FACS was performed by using a BD LSR Fortessa X-20 machine SORP (version 8.0.1) in the Janis V. Giorgi Flow Cytometry Core Facility, UCLA. For analysis and computational compensation of the data, BD FACS Diva software was used. Only live and singlet cells were gated to analyze Ep-CAM-positive cells; dead cells were excluded. Percent purity was determined to be ∼83%. Because ∼17% of the cells did not stain positively for CD326 (Ep-CAM), to rule out contaminating leukocytes or macrophages, we also performed IHC as previously described (14, 15) for markers CD8, LY6G, and F4/80, none of which were positive. The nature of the ∼17% of cells negative for CD326 is not clear.

#### Determination of IFN-β in enterocytes by ELISA.

The enterocytes were resuspended with 2.0 ml of a saline solution containing protease inhibitors (Roche Complete Mini, 1 tablet per 10 ml). The enterocytes were disrupted with a Fisher model 60 Sonic Dismembrator set at 5 watts for a duration of 2 s each time for five times. A 0.5 ml aliquot of the sonicate was brought to 6.25 ml by using the ELISA plate coating buffer, which was 50 mM sodium bicarbonate solution (pH 9.6). One milliliter of the resulting solution contained the equivalent of sonicated enterocytes from 2 mg of jejunum. The solution was diluted to 1:400 by using the bicarbonate buffer, and 100 μl was added to each well of a Costar flat-bottom high-binding Polystyrene Stripwell Microplate (catalog no. 2592). After incubation overnight at 4°C, the solution in the wells was aspirated, and the plates were washed four times with PBS and blocked with 1% BSA-PBS for 1 h at RT. The solution was then aspirated; the primary antibody anti-IFN β (Thermo Scientific, PA5-20390) at 1:500 dilution in 1% BSA-PBS was added; and the plates were incubated at 37°C for 2 h. The solution was aspirated, and the plates were washed four times with PBS. The secondary antibody, donkey anti-rabbit IgG-HRP (Jackson Immunolabs, catalog no. 711-035-152) was added at 1:5,000 dilution in 1% BSA-PBS and incubated at RT for 90 min with gentle mixing. The plates were then washed five times with PBS, and 3,3′,5,5′-tetramethylbenzidine substrate (KPL Labs, catalog no. 507600) was applied and gently mixed. The reaction was stopped by the addition of 3 M sulfuric acid, and the plates were read at 450 nm in a Molecular Devices Spectra MAX 190. The concentration of IFN-β was determined from a standard curve prepared with recombinant mouse IFN-β. The results shown are the mean ± SEM of IFN-β (nanograms per milliliter sonicate) from the enterocytes taken from the jejunum of eight individual mice for each treatment condition that were chosen at random from 24 mice subjected to each treatment condition.

#### Determination of CH25H in enterocytes by ELISA.

Enterocytes were prepared as described above, and CH25H ELISA was performed following the same protocol as for IFN-β ELISA, except for using a 1:3,200 dilution of the enterocyte solution and a 1:1,000 dilution of the antibody to CH25H purchased from Bioss Antibodies (catalog no. bs-6480R). The optical density value for each mouse was normalized to the mean value of the chow group, which was the lowest of the six treatment groups. The results shown are the mean ± SEM for the jejunums taken from 16 individual mice chosen at random from 24 mice for each treatment condition.

#### Determination of mRNA levels by quantitative RT-PCR for IFN-α, IFN-β, and CH25H in duodenum and jejunum.

The duodenum and jejunum were harvested after the mice were perfused to remove all blood (10, 16); mRNA was extracted, and quantitative RT-PCR (RT-qPCR) was performed as described previously (16).

#### Determination of 25-OHC in jejunum by LC-MS/MS.

Quantitation of 25-OHC was determined in jejunum samples that had been stored at −80°C. Water [double-distilled H_2_O (ddH_2_O), 200 μl] plus chloroform/methanol (2:1, 1,800 μl) containing 0.01% butylated hydroxytoluene, and 500 pmol of an internal standard (^2^H_6_-25-OHC, Avanti Polar Lipids, catalog no. 700053) was added to 100 mg of tissue sample from each of 16 randomly selected mice in each treatment group. After vigorous mixing, the samples were homogenized (IKA T10 Ultra-Turrax, 60 s) and then centrifuged (1,500 *g*, 15 min, RT). One milliliter was removed from the bottom (chloroform) phase and transferred to a new glass tube. To this, 1,100 μl of a solution consisting of methanol/ddH_2_O/formic acid (0.3/0.75/0.05 vol, respectively) was then added. After vigorous mixing and centrifugation (1,500 *g*, 15 min, RT), the bottom phase was transferred to a clean tube, overlayered with argon, and capped. The sample was then dried under a stream of argon, suspended in isopropyl alcohol (20 μl), mixed vigorously (30 s), and then placed on ice. Aqueous ammonium acetate (100 μl, 50 mM) containing 6 μg of cholesterol oxidase (Sigma-Aldrich, catalog no. C8649) was then added, and the samples were incubated (1 h, 37°C), after which the samples were dried again under an argon stream. Amplifex Keto Reagent (25 μl, Sciex catalog no.4465962) was then added, and the samples ware incubated (RT, 1 h) with mixing. The derivatized sample was stored at −80°C until analysis. For analysis, the samples were dried under vacuum and then suspended in 50 μl of methanol/ddH_2_O (70/30, v/v) and centrifuged (1,500 *g*, 15 min), and the supernatants were transferred to HPLC injector vials. Aliquots (8 μl) were injected onto a reverse-phase HPLC column (Imtakt Cadenza CD-C18, 250 × 2 mm, 3 μm particle size) equilibrated in eluent A (water/formic acid, 100/0.1, v/v) and eluted (200 μl/min) with an increasing concentration of eluent B (acetonitrile/formic acid, 100/0.1, v/v: min/% B; 0/30, 5/30, 50/100, 53/30, 60/30). The effluent from the column was connected to an electrospray ion source attached to a triple-quadrupole mass spectrometer (Agilent 6460) scanning in the positive multiple reaction monitoring (MRM) mode with standard resolution settings (full-width at half-maximum 0.7) by using previously optimized conditions for the following transitions: 25-OHC, *m/z* 515→456, corresponding to the transition of the M^+^ ions of the O-[3-(trimethylammonium) propyl] oxime derivatives to the resulting (M-N(CH_3_)_3_)^+^ fragments; ^2^H_6_-25-OHC, *m/z* 521→462. For construction of a standard curve, standard samples were prepared with authentic 25-OHC with the same amount of internal standard. Estimation of the amount of 25-OHC in each biological sample was made by interpolation from the standard curve (ordinate, ratio of 25-OHC/internal standard; abscissa, amount of OHC in each standard sample). Quantitation was achieved by using an Agilent MassHunter Workstation. Authentic 27-hydroxycholesterol (Avanti Polar Lipids, catalog no. 700021) served as a control. The derivatives of 25- and 27-OHC appeared as closely eluting, but distinct, bimodal peaks, presumably corresponding to the synisomer and antiisomer of each compound. The area of the entire bimodal peak in the MRM 515→456 trace for 25-OHC was measured and used in the calculations.

#### Determination of fecal neutral sterols.

LDLR-null mice were switched from chow to WD. After 2 weeks, the mice were switched back to chow containing a nonabsorbable color marker [0.05% by weight of Allura red AC (Sigma-Aldrich, catalog no. 458848)]. Forty-eight hours after the marker appeared in the feces, the mice were continued on chow alone, or Tg6F was added to the chow at 0.06% by weight of diet, or ezetimibe was added to the chow at 10 mg/kg/day, or ezetimibe and Tg6F prepared by the novel method were added to the chow to give ezetimibe 10 mg/kg/day and Tg6F at 0.06% by weight of diet. After 1 week, while still on these diets, the feces were collected, and fecal neutral sterols (FNS) were determined as described (9).

Plasma lipids and SAA levels were determined as previously described (8, 14, 15).

### Calculations and statistical analysis

To calculate the decrease or increase of a parameter after addition to WD of an agent(s), the difference between the average value for the parameter in mice on WD alone and the value for each mouse receiving the agent(s) was determined to give the decrease or increase compared with WD. Statistical analyses were performed initially by ANOVA. After determining that statistically significant differences were present by ANOVA, further comparisons were made by unpaired two-tailed *t*-test. All statistical analyses were performed by using GraphPad Prism version 5.03 or 7.02 (GraphPad Software, San Diego, CA). Statistical significance was considered achieved if *P* < 0.05. We compared adjacent bars in the figures by unpaired two-tailed *t*-test. If a pair of adjacent bars were not significantly different, but there was a significant difference compared with the next bar, in general, the *P* values for either of the bars that did not differ from each other compared with the next bar were the same, and in every case, the *P* value was significant. Therefore, to reduce clutter, in general, we only show the *P* values between adjacent bars in the figures.

## RESULTS

### Addition of either ezetimibe or Tg6F to WD ameliorated WD-induced dyslipidemia in LDLR-null mice; addition of both to WD (combined formulation) was more efficacious than either agent alone

**Fig. 1.**
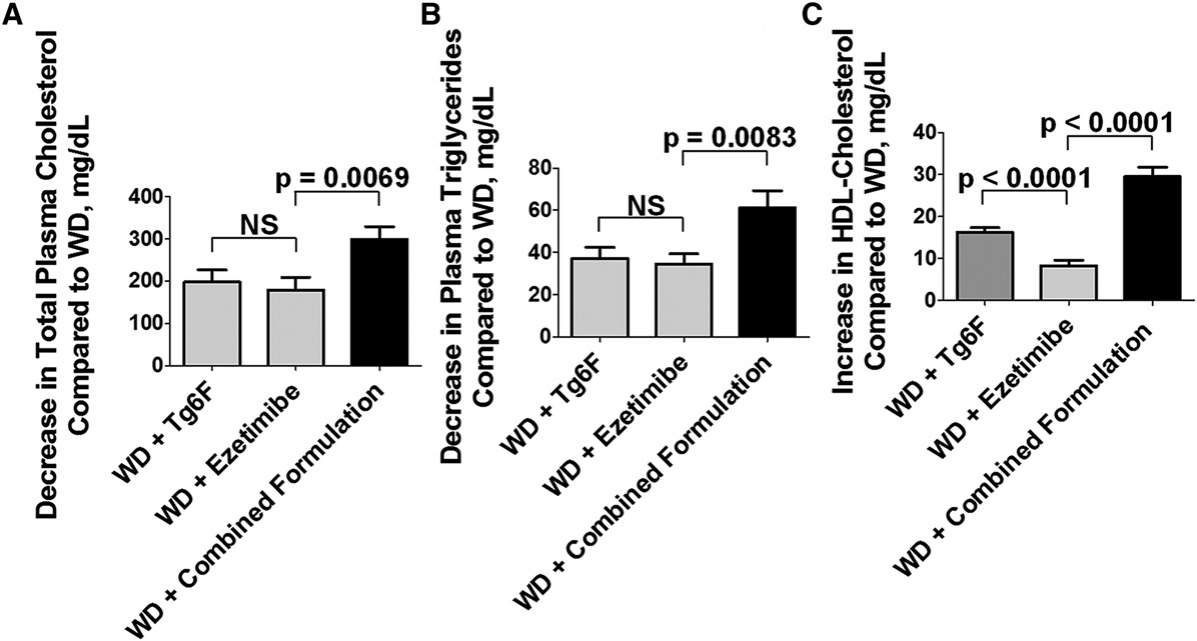
Addition of either ezetimibe or Tg6F to WD ameliorated dyslipidemia in LDLR-null mice and addition of both to WD (combined formulation) were significantly better than addition of either agent alone. Female LDLR-null mice age 3–6 months (n = 20–28 per group) were fed standard mouse chow or WD or the mice were fed WD plus 0.06% by weight of Tg6F concentrate, which was prepared as described in Materials and Methods; or the mice were fed WD with ezetimibe added to give a daily dose of 10 mg per kg of body weight (WD + ezetimibe); or the mice were fed WD with Tg6F added at 0.06% by weight plus ezetimibe (each added separately to the diet) to give a daily dose of ezetimibe of 10 mg per kg of body weight as described in Materials and Methods for the combined formulation. After feeding the diets for 2 weeks, the mice were bled, and plasma lipid levels were determined as described in Materials and Methods. A: The decrease in plasma cholesterol compared with WD alone for each treatment. B: The decrease in plasma triglycerides compared with WD alone for each treatment. C: The increase in plasma HDL cholesterol compared with WD alone for each treatment. The data shown are mean ± SEM. NS, not significant.

We previously reported that a dose of 0.06% by weight of diet of the Tg6F concentrate was near maximal for ameliorating dyslipidemia in LDLR-null mice on WD (15). Therefore, we tested Tg6F added to WD at 0.06% by weight. In mice, the ED_50_ value for ezetimibe was reported to be 700 μg/kg/day (17). We tested ezetimibe at a dose of 10 mg/kg/day, a dose equal to or greater than the highest dose used in mice that we found in the literature (11, 13, 17–19). As shown in the experiment described in **Fig. 1**, adding either Tg6F or ezetimibe to WD ameliorated dyslipidemia, and adding both to WD at the same doses (combined formulation) further ameliorated dyslipidemia. The values (mean ± SEM) for plasma total cholesterol on chow and WD were 134 ± 5 and 966 ± 48 mg/dl, respectively (*P* < 0.0001). Figure 1A presents the data plotted to show the decrease in plasma cholesterol compared with the level on WD alone for each treatment. The values for plasma triglyceride levels on chow and WD were 28 ± 1 and 171 ± 8 mg/dl, respectively (*P* < 0.0001). Figure 1B presents the data plotted to show the decrease in plasma triglycerides compared with the level on WD alone for each treatment. The values for plasma HDL cholesterol levels on chow and WD were 76 ± 2 and 46 ± 1 mg/dl, respectively (*P* < 0.0001). Figure 1C presents the data plotted to show the increase in plasma HDL cholesterol compared with the level on WD alone for each treatment. In this experiment, the efficacy of Tg6F and ezetimibe was not different when administered as single agents, except for HDL cholesterol levels, where Tg6F was superior to ezetimibe as a single agent. In each case in Fig. 1, the combined formulation was substantially more efficacious than the single agents were.

In six of six experiments (the experiments shown in **Figs. 1** and ** 2** and four additional experiments that are not shown), adding either ezetimibe or Tg6F as single agents to WD improved plasma total cholesterol and triglyceride levels. In five of five experiments in which plasma HDL cholesterol was measured, adding either ezetimibe or Tg6F as a single agent to WD increased the levels.

**Fig. 2.**
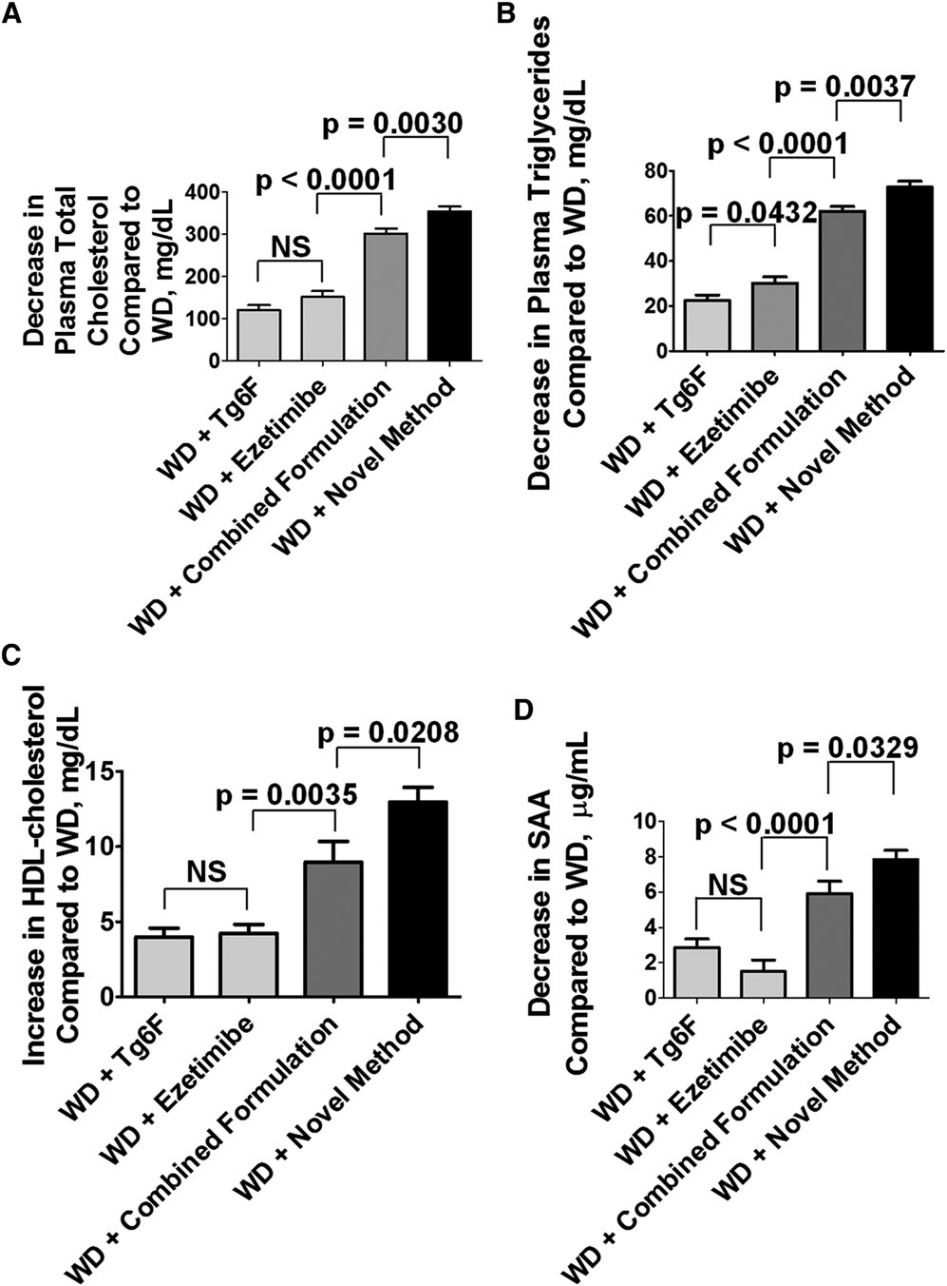
Adding Tg6F and ezetimibe to WD using the novel method was more effective than adding the same doses of Tg6F and ezetimibe to WD using the combined formulation. Female LDLR-null mice age 9–12 months (n = 25 per group) were fed standard mouse chow; or WD; or WD with Tg6F added at 0.06% by weight of diet (WD + Tg6F); or WD with ezetimibe added to give a daily dose of 10 mg per kg of body weight (WD + ezetimibe); or Tg6F added at 0.06% by weight of diet plus ezetimibe (each separately mixed into the diet) to give a daily dose of ezetimibe of 10 mg per kg of body weight (WD + combined formulation); or, by using the novel method as described in Materials and Methods, WD containing Tg6F at 0.06% by weight of diet and ezetimibe sufficient to provide the mice with 10 mg/kg/day (WD + novel method). After feeding the diets for 2 weeks, the mice were bled, the jejunums were harvested as described in Materials and Methods, and plasma lipid levels and plasma levels of SAA were determined. A: The decrease in plasma cholesterol compared with WD alone for each treatment. B: The decrease in plasma triglycerides compared with WD alone for each treatment. C: The Increase in plasma HDL cholesterol compared with WD alone for each treatment. D: The decrease in plasma SAA levels compared with WD alone for each treatment. NS, not significant.

In five of six experiments, the combined formulation was better than either agent alone in reducing plasma total cholesterol levels. In one experiment (data not shown), the mice receiving the combined formulation showed a trend for a decrease in plasma total cholesterol compared with the mice receiving the single agents, but the difference did not reach statistical significance.

In six of six experiments, the combined formulation was better than either agent alone in reducing plasma triglyceride levels.

In four of five experiments, the combined formulation was better than either agent alone in increasing plasma HDL cholesterol levels.

### A novel method for adding Tg6F an5d ezetimibe to WD that was more effective than the combined formulation

Instead of mixing Tg6F and ezetimibe separately into WD as in the combined formulation, we added ezetimibe during the preparation of the Tg6F concentrate. In making the Tg6F concentrate, freeze-dried tomato powder from transgenic 6F tomatoes was incubated at RT in ethyl acetate with 5% acetic acid (15). After an overnight incubation, the supernatant contained the 6F peptide, which was recovered by removing the ethyl acetate. The resulting solids were resuspended in water and subjected to a final freeze-drying that resulted in a uniform fluffy tomato powder that by weight was 37-fold more active than the starting material (14, 15). Surprisingly, we found that, after the overnight incubation, if we added ezetimibe to the ethyl acetate supernatant and incubated for 2 h at RT before removing the solvent, the final resulting lyophilized tomato powder containing ezetimibe and the 6F peptide was more effective. As shown in Fig. 2A–C, the novel method was more effective in ameliorating dyslipidemia compared with adding Tg6F or ezetimibe to WD as single agents or compared with the combined formulation. The values (mean ± SEM) for plasma total cholesterol on chow and WD were 164 ± 3 and 1,088 ± 17 mg/dl, respectively (*P* < 0.0001). Figure 2A presents the data plotted to show the decrease in plasma cholesterol compared with the level on WD alone for each treatment.

The values for plasma triglyceride levels on chow and WD were 37 ± 0.5 and 240 ± 4 mg/dl, respectively (*P* < 0.0001). Figure 2B presents the data plotted to show the decrease in plasma triglycerides compared with the level on WD alone for each treatment.

The values for plasma HDL cholesterol levels on chow and WD were 55 ± 1 and 40 ± 0.5 mg/dl, respectively (*P* < 0.0001). Figure 2C presents the data plotted to show the increase in plasma HDL cholesterol compared with the level on WD alone for each treatment.

The values (mean ± SEM) for plasma SAA on chow and WD were 7 ± 0.1 and 35 ± 0.5 μg/ml, respectively (*P* < 0.0001). Figure 2D presents the data plotted to show the decrease in plasma SAA compared with the level on WD alone for each treatment. In five of five experiments in which SAA was measured, adding ezetimibe or Tg6F as a single agent to WD decreased the levels. In four of five experiments, the combined formulation was better than either agent was alone in decreasing plasma SAA levels. In one experiment, the values for plasma SAA for the mice receiving the combined formulation were very slightly, but significantly, higher than the case for the single agents (data not shown).

In four of four experiments, despite having added to the diet the same amount of ezetimibe and Tg6F, plasma total cholesterol, plasma triglyceride, and plasma SAA levels were lower with the novel method compared with the administration of the single agents or the combined formulation. In three of three experiments in which HDL cholesterol was measured, plasma HDL cholesterol levels were higher with the novel method compared with the administration of the single agents or the combined formulation.

Similar results to those shown in Fig. 2 were found in male mice (data not shown).

### WD induced IFN-β, CH25H, and 25-OHC in jejunum

**Fig. 3.**
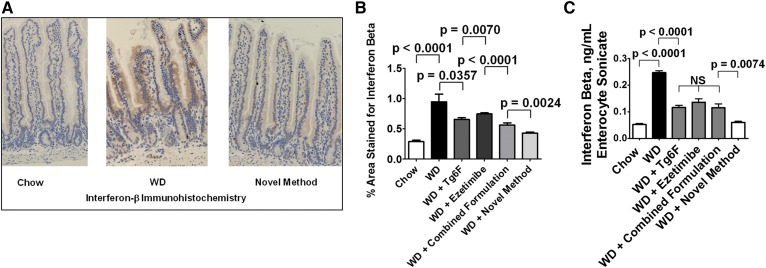
WD induced increased levels of IFN-β in the enterocytes of the jejunum of the mice described in Fig. 2, which was better ameliorated by the novel method for adding Tg6F and ezetimibe compared with adding the same doses of Tg6F and ezetimibe to WD by the combined formulation. The induction of IFN-β by WD in the enterocytes of the jejunum was determined by IHC and ELISA as described in Materials and Methods. A: An example of IHC showing induction of IFN-β expression by WD and amelioration by the novel method. B: Quantification of IHC was performed as described in Materials and Methods. C: The results of IHC were confirmed by ELISA as described in Materials and Methods. The results shown are mean ± SEM. Abbreviations are the same as in the Fig. 2 legend.

The data in **Fig. 3A, B** demonstrate that WD increased the expression of IFN-β in the enterocytes of the jejunum as shown by IHC. The IHC results for IFN-β in Fig. 3B are expressed as the percent area of the intestinal villus that was positively stained for IFN-β. The IHC results for IFN-β were confirmed by ELISA as shown in Fig. 3C.

**Fig. 4.**
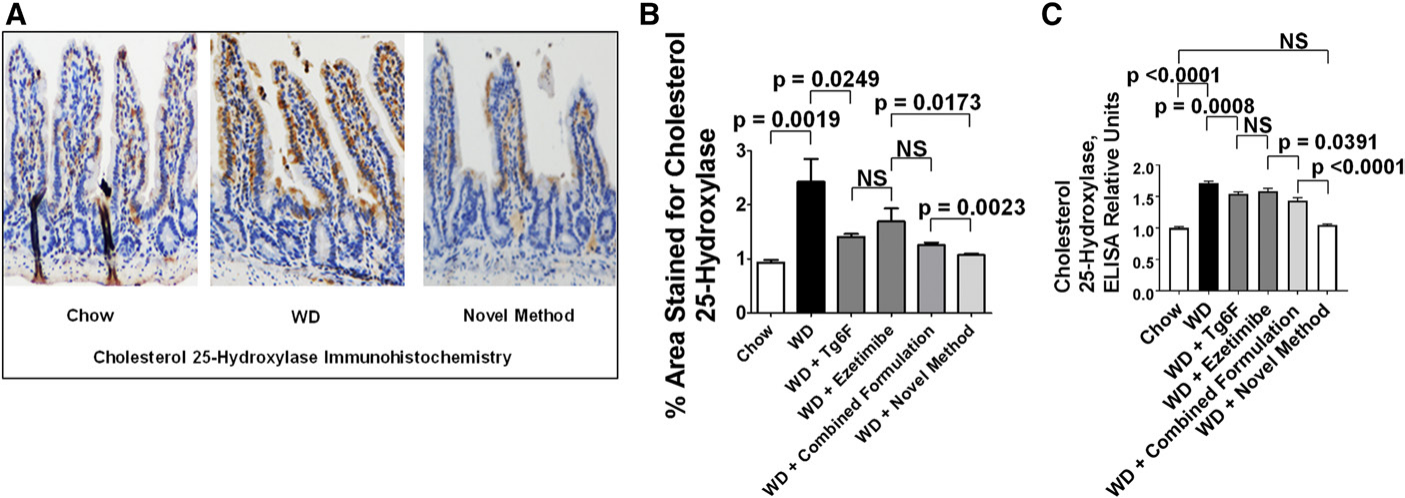
WD increased levels of CH25H in the enterocytes of the jejunum of the mice described in Fig. 2, which was better ameliorated by the novel method for adding Tg6F and ezetimibe compared with adding the same doses of Tg6F and ezetimibe to WD by the combined formulation. The induction of CH25H by WD in the enterocytes of the jejunum was determined by IHC and ELISA as described in Materials and Methods. A: An example of IHC showing induction of CH25H expression by WD and amelioration by the novel method. B: Quantification of IHC was performed as described in Materials and Methods. C: The results of IHC were confirmed by ELISA as described in Materials and Methods. The results shown are mean ± SEM. Abbreviations are the same as in the Fig. 2 legend.

IFNs are known to induce the expression of CH25H (20). Consistent with the induction of IFN-β by WD, **Fig. 4A, B** demonstrates that WD also increased the expression of CH25H in the enterocytes of the jejunum as shown by IHC. The IHC results for CH25H in Fig. 4B are expressed as the percent area of the intestinal villus that was positively stained for CH25H. The IHC results for CH25H were confirmed by ELISA as shown in Fig. 4C.

**Fig. 5.**
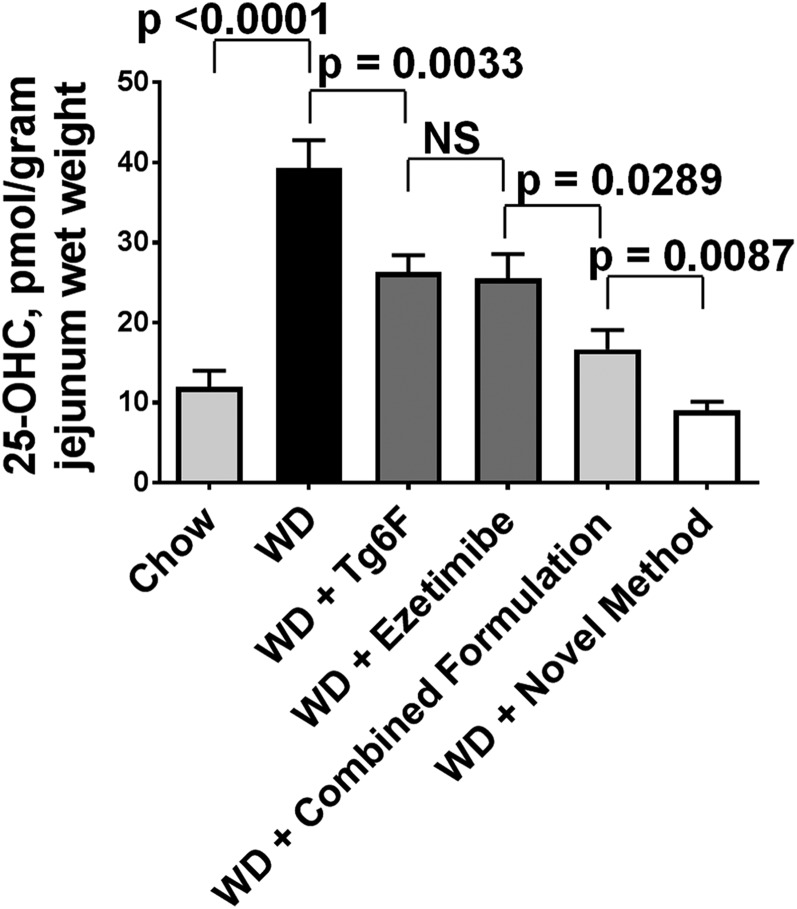
WD induced increased levels of 25-OHC in the jejunum of the mice described in Fig. 2, which was better ameliorated by the novel method described in Materials and Methods for adding Tg6F and ezetimibe compared with adding the same doses of Tg6F and ezetimibe to WD by the combined formulation. The 25-OHC was determined by LC-MS/MS as described in Materials and Methods. The results shown are mean ± SEM. Abbreviations are the same as in the Fig. 2 legend.

With an increase in the expression of CH25H protein levels in jejunum, we would expect an increase in the levels of 25-OHC in jejunum. The data in **Fig. 5** indeed demonstrate that feeding WD resulted in increased levels of 25-OHC in jejunum, which were ameliorated by Tg6F or ezetimibe added as single agents or added together as the combined formulation or by the novel method. The data in Figs. 4 and 5 and in an additional experiment (data not shown) indicate that the novel method was superior to the combined formulation in decreasing the levels of IFN-β, CH25H, and 25-OHC in the jejunum.

#### WD induced mRNA levels for IFN-α, IFN-β, and CH25H in duodenum and jejunum.

**Fig. 6.**
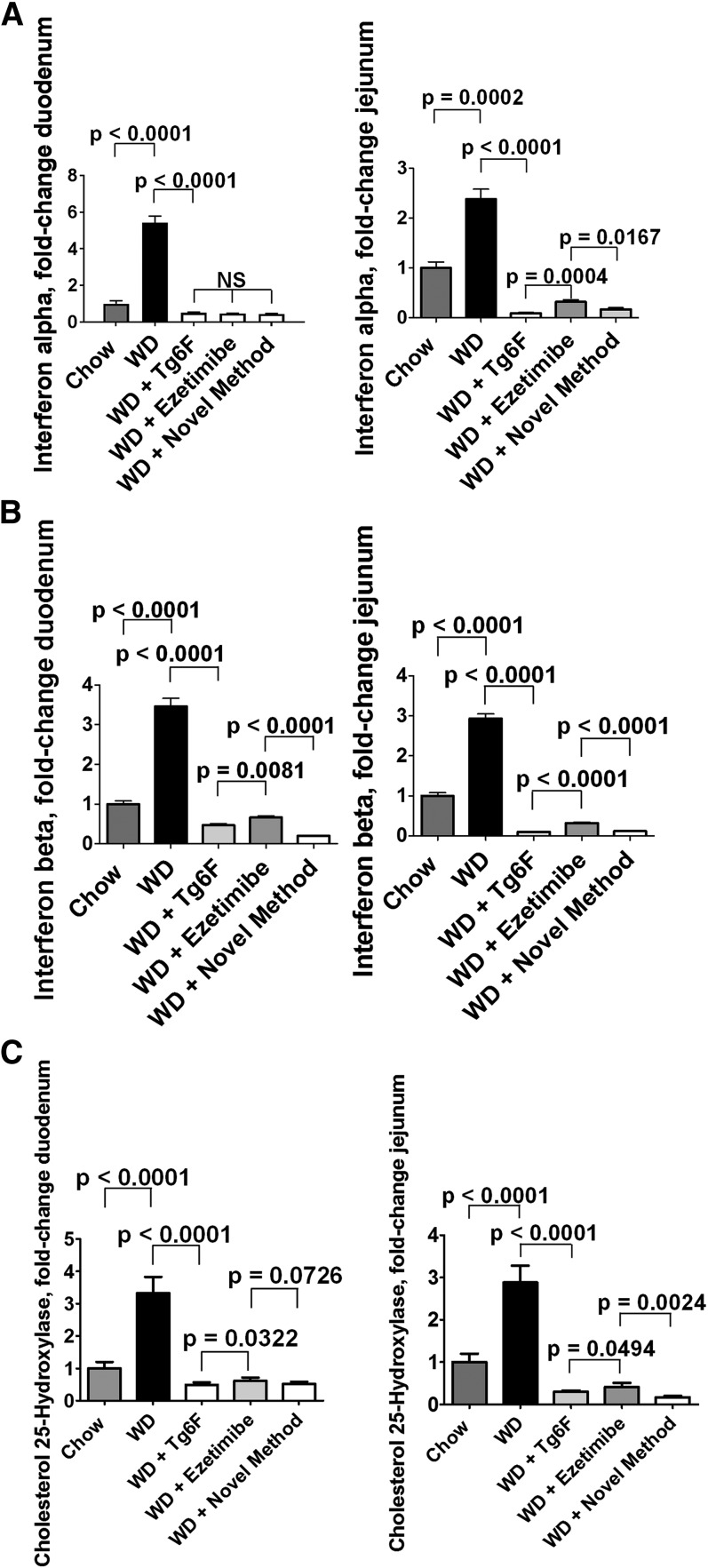
Feeding LDLR-null mice WD resulted in increased mRNA levels for IFN-α, IFN-β, and CH25H in the duodenum and jejunum of the mice described in Fig. 2, and adding Tg6F and ezetimibe to WD prevented the increase. IFN-α, IFN-β, and CH25H mRNA levels in the duodenum and jejunum were determined by RT-qPCR as described in Materials and Methods. A: Results for IFN-α are shown. B: Results for IFN-β are shown. C: Results for CH25H are shown. In each case, the left panel shows the results for the duodenum, and the right panel shows the results for the jejunum. The results are mean ± SEM. Abbreviations are the same as in the Fig. 2 legend.

We previously reported that WD-mediated changes in the expression of a number of genes as determined by RT-qPCR were similar in the duodenum and jejunum, as was the change in the expression of these genes after adding Tg6F to WD (16). The data in **Fig. 6** demonstrate that this was also the case for IFN-α (Fig. 6A), IFN-β (Fig. 6B), and CH25H (Fig. 6C). The data demonstrate that the induction of IFN-α and IFN-β were similar, and these genes responded similarly to adding Tg6F and ezetimibe to WD as single agents or adding them together by the novel method.

#### Addition of Tg6F or ezetimibe as single agents to chow or adding both to chow via the novel method increased FNS excretion.

**Fig. 7.**
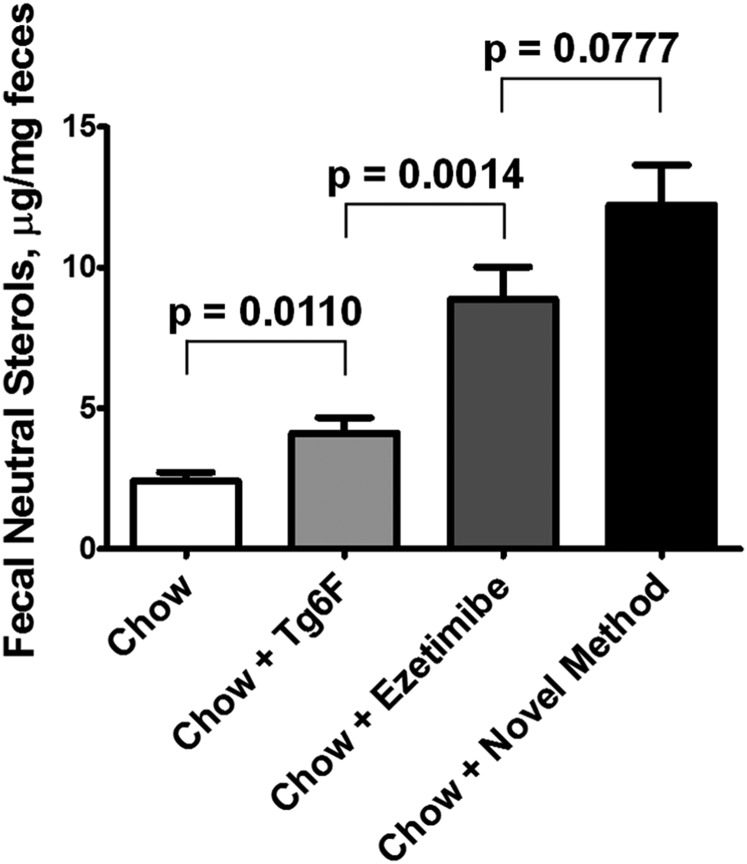
Addition of Tg6F or ezetimibe as single agents to chow or adding both via the novel method significantly increases FNS excretion. Female LDLR-null mice were housed four mice per cage, and three to five cages per group were used in each of three separate experiments. The mice were ages 3–4, 9–12, and 14–15 months, respectively, for the three experiments. In each experiment, the mice were switched from chow to WD. After 2 weeks, the mice were switched back to chow containing a nonabsorbable color marker as described in Materials and Methods. Forty-eight hours after the marker appeared in the feces, the mice were continued on chow alone (chow) or Tg6F was added to the chow at 0.06% by weight of diet, or ezetimibe was added to the chow at 10 mg/kg/day, or ezetimibe and Tg6F prepared by the novel method were added to the chow to give Tg6F at 0.06% by weight of diet and ezetimibe 10 mg/kg/day. After 1 week, while still on these diets, the feces were collected from each cage, and FNS was determined as described in Materials and Methods. Shown are mean ± SEM of the pooled data from the three separate experiments.

As shown in **Fig. 7**, adding Tg6F or ezetimibe to WD as single agents or adding both to WD via the novel method significantly increased FNS excretion. However, in contrast to all of the parameters measured in Figs. 1–6, ezetimibe was significantly more effective than Tg6F in increasing FNS. Moreover, in contrast to the parameters measured in Figs. 4–6, the increase in FNS by the novel method compared with ezetimibe alone did not quite achieve statistical significance.

## DISCUSSION

To our knowledge, this is the first report that IFN-β is induced in the enterocytes of the small intestine by WD, and this is the first report that small intestine IFN-β, CH25H, and 25-OHC levels are potently and favorably modulated by an oral apoA-I mimetic peptide and ezetimibe.

WD increased the expression of IFN-β protein (Fig. 3) and CH25H protein (Fig. 4). With the increase in CH25H, there was a corresponding increase in 25-OHC levels in jejunum, as shown in Fig. 5. Adding ezetimibe or Tg6F to WD as single agents or in the combined formulation or by using the novel method resulted in changes in 25-OHC levels that paralleled the changes in IFN-β and CH25H protein. For example, the levels of these proteins on WD with Tg6F and ezetimibe added by the novel method were either slightly, but significantly, lower (IFN-β) or not different (CH25H) from the levels on chow. Similar results were seen for the levels of 25-OHC in jejunum; the levels of 25-OHC in jejunum of mice on WD with Tg6F and ezetimibe added by the novel method were not different from the levels on chow. Although treatment with the novel method essentially normalized the levels of IFN-β, CH25H, and 25-OHC, this treatment only partially ameliorated dyslipidemia and systemic inflammation as measured by plasma SAA levels. Thus, IFN-β, CH25H, and 25-OHC levels in the small intestine can explain only a portion of the dyslipidemia and systemic inflammation that results from feeding WD to LDLR-null mice.

As indicated in Results, the reproducibility of our findings between experiments was very strong from a directional view point (e.g., in five of five experiments in which plasma HDL cholesterol was measured, adding either ezetimibe or Tg6F as a single agent to WD increased plasma HDL cholesterol levels. In four of five experiments, the combined formulation was better than either agent alone in increasing plasma HDL cholesterol levels. In three of three experiments in which HDL cholesterol was measured, plasma HDL cholesterol levels were higher with the novel method compared with the administration of the single agents or the combined formulation). However, in the examples given in the figures, there was considerable variation in the baseline values for HDL cholesterol levels and in the response to therapy. For example, in Fig. 1C, the values for plasma HDL cholesterol levels on chow and WD were 76 ± 2 and 46 ± 1 mg/dl, respectively (*P* < 0.0001). In Fig. 2C the values for plasma HDL cholesterol levels on chow and WD were 55 ± 1 and 40 ± 0.5 mg/dl, respectively (*P* < 0.0001). The magnitude of the change achieved by adding Tg6F or ezetimibe or the combined formulation to WD also varied. The mice in Fig. 1C were 3–6 months old, and in Fig. 2C, the mice were 9–12 months old; perhaps the age differences contributed to the variation. Regardless, these data emphasize the importance of using sufficient numbers of mice and of repeating the experiments a sufficient number of times to have confidence in the results.

Feeding LDLR-null mice a chow diet supplemented with unsaturated (but not saturated) lysophosphatidylcholine (LysoPC) or lysophosphatidic acid (LPA) produced dyslipidemia and systemic inflammation (plasma SAA and IL-6 levels), and the extent and cellular characteristics of aortic atherosclerosis approached that seen on WD (14, 16, 21). Perhaps the levels of IFN-β, CH25H, and 25-OHC in the small intestine only account for a portion of the unsaturated LysoPC and LPA produced by feeding WD to LDLR-null mice.

**Fig. 8.**
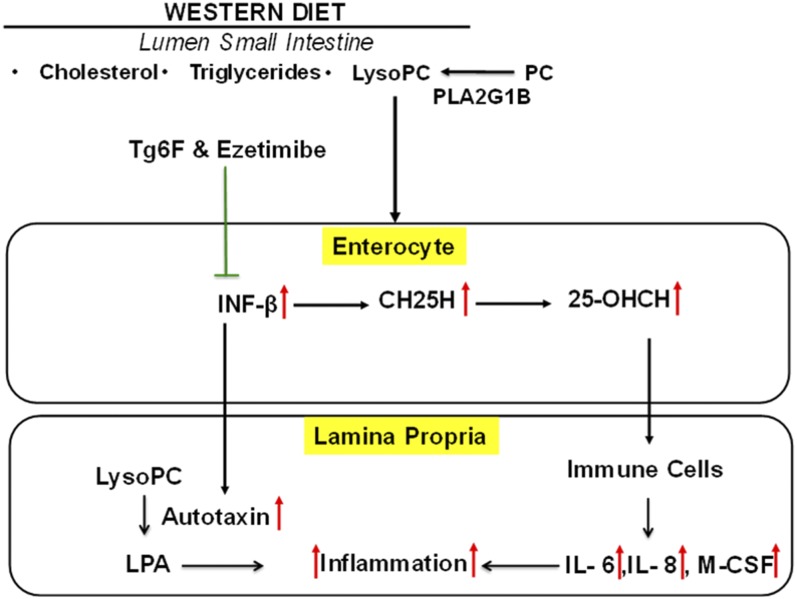
A hypothesis to explain how WD induces inflammation in the jejunum that Tg6F and ezetimibe ameliorate. WD provides cholesterol, triglycerides, and phospholipids [phosphatidylcholine (PC)]. In the lumen of the small intestine, phospholipase A_2_ group 1B (PLA2G1B) removes the fatty acid moiety from the *sn*-2 position of PC, yielding LysoPC. The triglycerides are acted upon by pancreatic lipase to yield two fatty acids and a monoglyceride (data not shown). Also not shown is the action of bile in forming micelles. The enterocytes of the small intestine take up the dietary lipids together with small amounts of ligands for TLRs. This induces IFN-β in the enterocytes, which in turn induces CH25H, which contributes to increased levels of 25-OHC in the enterocytes. Subsequently, 25-OHC enters the lamina propria of the villi of the small intestine, where it acts on immune cells. This leads to increased levels of inflammatory cytokines [e.g., 25-OHC is known to induce increased levels of IL-6, IL-8 (mouse functional homologs MIP2 and KC), and M-CSF], which induce inflammation in the villus of the small intestine. Additionally, IFN-β can induce the expression of autotaxin that increases the conversion of unsaturated LysoPC to unsaturated LPA, which further stimulates inflammation and leads to dyslipidemia as discussed in refs. 14, 16, and 25. Intestinal inflammation leads to systemic inflammation as measured by increased levels of plasma SAA (data not shown). Ezetimibe and Tg6F act to decrease levels of IFN-β, CH25H, and 25-OHC, thus partially ameliorating inflammation and dyslipidemia in LDLR-null mice fed WD.

IFN-β is a potent inducer of CH25H, which broadly inhibits viral entry (20). CH25H and its product 25-OHC have both antiinflammatory properties, such as inhibiting the conversion of pro-IL-1β to IL-1β, and proinflammatory properties, such as inducing the expression of IL-6, IL-8, and macrophage colony-stimulating factor (M-CSF) (22). Additionally, IFN-β has been reported to be a potent autocrine and paracrine factor that induces autotaxin in response to Toll-like receptor (TLR) activation (23). We have previously reported that WD contains little unsaturated LysoPC or unsaturated LPA, but potently induces increased levels of both unsaturated LysoPC and unsaturated LPA in the small intestine and in plasma (10, 14, 16). Others have reported that a high-fat diet led to increased endotoxin levels in intestine and plasma of mice (24), and a Western-style diet led to a 71% increase in plasma levels of endotoxin in humans (25). Thus, we would expect WD to increase TLR ligands in the tissue of the small intestine where fat is absorbed (primarily in the duodenum and jejunum). If autotaxin were induced under these conditions, we would expect increased conversion of unsaturated LysoPC to unsaturated LPA (21), which would cause increased inflammation (14, 16). LysoPC stimulates IFN-β production in cultured cells (26). Because we previously demonstrated that WD increases the levels of LysoPC in the small intestine (14), this may in turn contribute to the increased expression of IFN-β. We focused on IFN-β in our studies, because of the availability of high-quality commercially available reagents, and because others have shown that IFN-α and IFN-β are similar in their ability to induce CH25H (27). As shown in Fig. 6, the induction of increased mRNA levels in the duodenum and jejunum by WD was similar, as was the response to adding Tg6F and ezetimibe to WD. Figure 6 also shows that at the level of mRNA, IFN-α and IFN-β responded similarly in both duodenum and jejunum. These results are consistent with our previous report that the WD-mediated change in the expression of a number of genes and their response to adding Tg6F to WD was similar in duodenum and jejunum (16). The response to adding Tg6F or ezetimibe or adding both by the novel method to WD resulted in mRNA expression that was below that of chow (Fig. 6). Measurements of IFN-β protein (Fig. 3), CH25H protein (Fig. 4), and 25-OHC mass (Fig. 5) showed significant, but less dramatic, reductions for all treatments except for the novel method, which resulted in reductions to levels about or below that seen on chow. The reason(s) for these differences is not known. **Figure 8** depicts the flow of “information” from the lumen to the enterocytes to the lamina propria of the villi. It is likely that this is an oversimplification and that events in the lamina propria feedback and alter the response of the enterocytes. Further research will be required to elucidate these details.

We would expect the increased levels of 25-OHC to stimulate liver X receptor (LXR). Although we did not measure LXR in these studies, it is known that increasing LXR activity in the intestine leads to decreased NPC1L1 and ACAT2 expression and increased expression of ABCG5/G8 and ABCA1, all of which would tend to mitigate the increased cholesterol burden of WD (28). Despite these expected offsetting changes, lipid levels remained high in the LDLR-null mice on WD, and SAA levels remained elevated. Adding Tg6F and ezetimibe to WD appears to tip the balance in favor of reduced lipid levels and reduced SAA levels, but even these agents do not completely ameliorate the effects of WD in LDLR-null mice.

Ezetimibe promotes TICE by targeting NPC1L1 protein, which blocks internalization of cholesterol from the brush border membrane, causing cholesterol in the brush border membrane to exit by diffusion into the lumen of the small intestine (11) or be pumped out into the lumen of the small intestine by ABCG5/ABCG8 (12).

We recently reported a remarkable affinity of the apoA-I mimetic peptide 4F for the duodenum and jejunum in mice after injection of the peptide into the tail vein (9). Both the 4F peptide and ezetimibe have been reported to promote TICE (9, 11, 13). Both Tg6F and ezetimibe lower plasma cholesterol levels. Both Tg6F and ezetimibe promoted FNS excretion from the small intestine (Fig. 7). However, in contrast to all of the other parameters measured in Figs. 1–5 in which Tg6F as a single agent was as effective or was more effective than ezetimibe in ameliorating the effects of WD, ezetimibe was significantly more effective than Tg6F in promoting FNS excretion (Fig. 7). Moreover, in contrast to all of the parameters measured in Figs. 2–5, the increase in FNS in Fig. 7 by the novel method compared with ezetimibe alone did not quite achieve statistical significance. Based on these results, we conclude that increased FNS excretion cannot be the only determinant of the effectiveness of Tg6F, and, indeed, it may not be the major determinant of Tg6F’s effectiveness. It seems more likely that increased FNS excretion plays a major role (probably the major role) in the effectiveness of ezetimibe.

Having chosen for these experiments near-maximal doses of Tg6F based on our previously published data (15) and ezetimibe based on the literature (11, 13, 17–19), we hypothesize that Tg6F and ezetimibe have different mechanisms of action that functionally overlap. Further research will be required to determine the precise site and mechanism of action of Tg6F and the site and mechanism of action for the combination of Tg6F and ezetimibe prepared by the novel method.

A surprising finding in the present study was that adding ezetimibe to the supernatant containing ethyl acetate with 5% acetic acid during the preparation of the Tg6F concentrate resulted in a final preparation that, when added to WD, was more effective compared with the single agents or the combined formulation. What explains the increased effectiveness of the novel method? There are several possibilities. During the preparation of the final concentrate, there might be noncovalent (or, less likely, covalent) formation of a new molecule that is more effective. Another possibility is that during the preparation of the concentrate, the tomato polyphenols together with the 6F peptide form micelles, which ezetimibe inserts into, and results in better targeting of ezetimibe and Tg6F in the small intestine. Future research will be required to determine the mechanism, but the data are clear that the novel method results in a more active preparation, which may be due to the formation of an ezetimibe-Tg6F-associated peptide.

David Y. Hui (29) recently emphasized the importance of intestinal phospholipid and lysophospholipid metabolism in causing the inflammation associated with cardio-metabolic disease. Figure 8 schematically depicts a hypothesis for the ability of Tg6F and ezetimibe to ameliorate that portion of dyslipidemia and systemic inflammation that is due to WD-mediated increases in IFN-β, CH25H, and 25-OHC.

In Fig. 8, Tg6F and ezetimibe are shown as acting on the enterocyte. Li et al. (30) recently demonstrated the importance of gut microbiota in generating increased atherogenic lipid metabolites. Zhong et al. (31) reported that gut microbiota can regulate whole-body cholesterol homeostasis and its response to ezetimibe. Our data cannot exclude the possibility that some or all of the effects of Tg6F and ezetimibe are mediated by their actions on the gut microbiota. Future research will be required to test the hypothesis proposed in Fig. 8 and determine the precise mechanisms involved. Such future studies should directly test the effects of knocking down IFN-β or CH25H in the enterocytes of the small intestine in LDLR-null mice fed WD. We believe that the data in this article provide a strong rationale for such research.
